# Effects of menopausal hormone therapy on gut microbiota in postmenopausal women and the relationship with bone metabolism

**DOI:** 10.3389/fmed.2025.1682925

**Published:** 2025-10-27

**Authors:** Jieqi Xiong, Lijun Li, Meihong Ao, Yunxia Tu, Kaijia Tu, Longyu Li

**Affiliations:** ^1^Jiangxi Medical College, Nanchang University, Nanchang, Jiangxi, China; ^2^Gynecology Department, Jiangxi Provincial Maternal and Child Health Hospital, Nanchang, Jiangxi, China; ^3^Oncology Department, Jiangxi Provincial Maternal and Child Health Hospital, Nanchang, Jiangxi, China

**Keywords:** postmenopausal, MHT, bone metabolism, gut microbiota, osteoporosis

## Abstract

**Objectives:**

Menopausal Hormone Replacement Therapy (MHT) is widely used by peri- and post-menopausal women to alleviate menopause-related symptoms and preventing bone loss, but the underlying mechanisms remain inadequately elucidated. Accumulating evidence suggested that gut microbiota was involved in the regulation of bone metabolic processes. The aim of this study was to characterize the alterations in gut microbiota profiles by MHT treatment in postmenopausal women and explore the relationship between gut microbiota and bone metabolism.

**Methods:**

Fecal samples collected from a total of 31 postmenopausal women with or without MHT were subjected to 16S ribosomal RNA (rRNA) gene sequencing and short-chain fatty acid (SCFAs) analysis in this study. The serum levels of bone metabolic markers were determined via chemiluminescent immunoassays. Spearman correlation coefficient was utilizes to assess the correlation between genera and bone metabolism indexes.

**Results:**

Postmenopausal women undergoing MHT exhibited lower serum procollagen type I N propeptide (P1NP) and C-terminal telopeptide of type I collagen (CTX-1). Significant differences in alpha diversity and beta diversity were observed in the microbial compositions between two groups (*P* < 0.05). Of the total 295 microbial taxa identified, 16 taxa displayed significant differential abundance, with Coprococcus, Eubacterium_ruminantium_group, Lachnospiraceae_UCG-010 being more enriched in MHT+, correlating with lower bone metabolic markers and higher estrogen level. Conversely, Escherichia-Shigella taxa was more abundant in MHT- group, positively correlating with high bone metabolic markers and lower estrogen level. SCFAs appeared to have a limited role in bone metabolism but were found to be associated with several genera, including Coprococcus, Adlercreutzia, Colidextribacter.

**Conclusions:**

The findings of the study demonstrated that MHT has the potential to prevent osteoporosis through the alteration of the gut microbial composition in postmenopausal women and identified promising microbial taxonomic that may contribute to the protective effects of MHT on bone mass conservation. Comparing with most previous studies that focused on the gut microbiota profiles between individuals with different bone mass, our study emphasized the protective role of gut microbiota in MHT process while bone mineral content (BMC) has no significant difference.

## Introduction

Osteoporosis is a progressive systemic skeletal disease characterized by reduced bone mass and microarchitectural alteration in bone tissue. Estrogen facilitates gut injury repair. Impairment of this function in low-estrogen states, such as postmenopause, may compromise intestinal barrier integrity, resulting in increased gut permeability. This promotes bacterial translocation—the dissemination of gut microbiota to extraintestinal sites via the bloodstream—potentially elevating the risk of metabolic and systemic diseases (1). The deficiency of endogenous estrogen after menopause leads women to bone loss, which increase the susceptibility to osteoporosis and bone fractures (2). As the lifespan of women extends in contemporary times, it is estimated that ~10% of the world's population and over 30% of postmenopausal women aged over 50 years suffered from osteoporosis according to statistics worldwide. Healthcare practitioners, including clinicians, dietitians, sports specialist are devoting much concerns to this issue.

Menopausal Hormone Replacement therapy (MHT) is a proven intervention for the prophylaxis of postmenopausal osteoporosis, which is recommended by International Menopause Society as a principle measures to improve the quality of life (3). The administration of MHT involves one to three categories of hormones: estrogens, the primary active constituent; progestogens, protecting endometrium against cancer in women with a uterus; and androgens when is needed. The underlying mechanisms by which MHT influences bone mass are complex, revealed factors include inflammation, immune response, intestinal mucosal barrier function and so on, while some are still unknown. Despite the numerous are there, the utilization of MHT is limited because of the potential risks for adverse events and uncertainty for prelonged hormone use. A more profound and comprehensive understanding of MHT is imperative.

Gut microbiota, known as the total microorganisms residing within the gastrointestinal tract, differs with gender, age, healthy statue and hormone level. Significantly, the gut microbiota contributes to numerous chronic diseases (4). Accumulating studies suggested that gut microbiota linked to bone metabolism and the maintenance of bone homeostasis, involving the formation by osteoblasts and resorption by osteoclasts through the so-called “gut microbiota-bone” axis (5). Previous studies have shown that postmenopausal osteoporosis is associated with diminished bacterial richness and diversity, as well as significant changes in abundance levels among phyla and genera in the microbial compositions (6, 7). Postmenopausal osteoporosis (PMO), also known as primary type I osteoporosis, is characterized by high transition rate, which is different from senile osteoporosis. Bone turnover markers (BTMs) serve as biomarkers for the diagnosis and evaluating the bone metabolic state (8). Therefore, it is of great significance to study PMOs separately and explore the relationship between gut microbiota and bone metabolism.

Microbiota plays an important role in sex steroid deficiency-associated bone loss (9). The relationship between bone metabolism and gut microbiota in MHT for postmenopausal women remains underexplored. Most previous studies have focused on comparing the gut microbiota profiles between individuals with different bone mass. In this study, we intended to investigate the relationship between MHT and gut microbiota in postmenopausal women with similar BMC, emphasize on bone metabolism from the perspective of the microbiota, while gut microbiota would be a new target for the prevention and treatment of PMO.

## Materials and methods

### Participants recruitment

**Figure 1:**
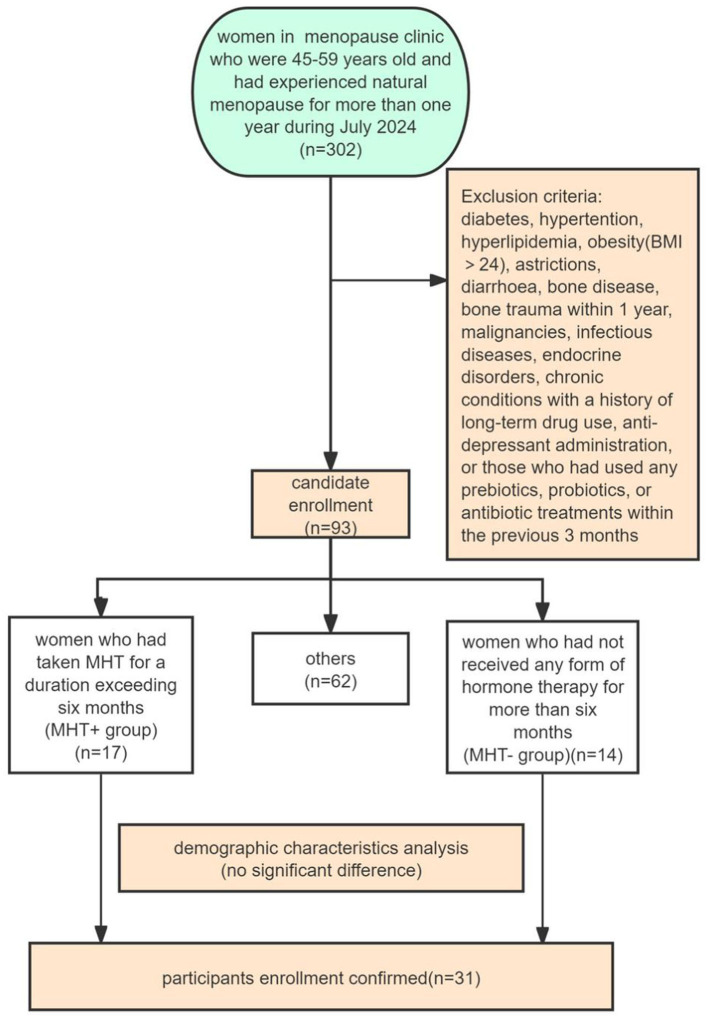
Participants enrollment flowchart.

The project was permitted by ethical committee of Jiangxi provincial maternal and children hospital before carrying out (EC-KY-2024098), written informed consent was obtained from all participants before enrollment. Participants were recruited from Jiangxi provincial maternal and children hospital and all residing in Jiangxi province. All participants had similar dietary habits. Women who were 45–59 years old and had experienced natural menopause for more than 1 year were eligible for inclusion in this study. Exclusion criteria comprised individuals with diabetes, hypertention, hyperlipidemia, obesity (BMI > 24), astrictions, diarrhea, bone disease, bone trauma within 1 year, malignancies, infectious diseases, endocrine disorders, chronic conditions with a history of long-term drug use, anti-depressant administration, any medications related to bone metabolism, or those who had used any prebiotics, probiotics, or antibiotic treatments within the previous 3 months. None of them had smoking history. The participants were divided into two groups: postmenopausal women who had taken hormone-replacement therapy for a duration exceeding 6 months (MHT+ group); postmenopausal women who had not received any form of hormone therapy for more than 6 months (MHT– group), Ultimately, 31 individuals who met all of these criteria and had used or not used MHT for more than half a year were enrolled in our study finally (*n* = 17 in the MHT+ group; *n* = 14 in the MHT– group) (Figure 1). Detailed information on hormone therapy, usage of alcohol, recreational drugs, travel history, nutritional supplements, physical exercising, menstrual and pregnancy histories were collected by questionnaire. All data were identified prior to analysis and all tests were completed during one morning.

### Clinical measurements

Height (m), weight (kg), circumferences (m) of the waist and hips, blood pressure were recorded for every participant. Body Mass Index (BMI) was calculated as weight/height^2^. A Dual-energy X-ray Absorptiometry (DXA) scanner (NORLAND, XR-600, US) was utilized for measuring Bone Mineral Density (BMD) (g/cm^2^) for the lumbar spine (LS, L1-4) and femoral neck (FN). The coefficients of variation (CV), as the precision indicator, were 0.9% and 1.4% for the spine and hip BMD measurements, respectively. BMD were recorded as the ratio of bone mineral content (BMC) (g) to bone area (cm^2^), and these data are presented as g/cm^2^. Biomarkers of Bone turnover (BTMs) are affected by circadian rhythmicity, with peak values occurring in the early morning and nadirs in the early afternoon and evening. Therefore, all venous blood samples were rigorously collected at similar time points in the morning to minimize these fluctuations. Fasting serum levels of BTMs, including osteocalcin (OC), serum C-terminal telopeptide of type I collagen (CTX-1), serum procollagen type I N propeptide (P1NP), parathyroid hormone (PTH), calcitonin (CT), and serum 25-hydroxyvitamin D3 (25(OH)VD3), were quantified with an automated Roche Osteoporosis Int electrochemiluminescence system (Roche Diagnostics GmbH, Germany). The CVs for quality control was determined to be within 4.01%−3.57% for osteocalcin, 4.04%−3.57% for CTX-1, 4.16%−4.01% for P1NP, 6.68%−4.42% for PTH, 4.53%−1.98% for CT and 3.21%−1.5% for 25(OH)VD3, for the high and low level controls, respectively. E2, Po, and T were quantified using a chemiluminescence immunoassay analyzer (DXI 800, Beckman Coulter, USA). The CVs for quality control were determined to be within 2.95%−2.01% for E2, 4.80%−6.21% for Po, 3.01%−3.93% for *T*, for the high and low level controls, respectively.

### Feces sample collection, 16S ribosomal RNA gene sequencing and data analysis

Human fecal samples was collected in sterile plastic containers, then the fecal samples were subsequently transferred for storage at −80 °C until use. Total microbial genomic DNA was extracted using the FastPure Stool DNA Isolation Kit (MJYH, shanghai, China) according to the manufacturer's instructions. The integrity and concentration of the extracted DNA were assessed by 1% agarose gel electrophoresis and a NanoDrop^®^ ND-2000 spectrophotometer (Thermo Scientific Inc., USA) and kept at −80 °C prior to further use. The extracted DNA was served as a template for amplifying the hypervariable V3-V4 regions of the bacterial 16S rRNA gene, utilizing the primer pairs 338F (5′-ACTCCTACGGGAGGCAGCAG3′) and 806R (5′-GGACTACHVGGGTWTCTAAT3′) by an T100 Thermal Cycler (BIO-RAD, USA). The PCR reaction mixture consisted of 4 μl of 5 × Fast Pfu buffer, 2 μl of 2.5 mM dNTPs, 0.8 μl of each primer (5 μm), 0.4 μl of Fast Pfu polymerase, 10 ng of template DNA, and ddH_2_O to achieve a final volume of 20 μl. PCR amplification cycling conditions were as follows: initial denaturation at 95 °C for 3 min, followed by 27 cycles of denaturing at 95 °C for 30 s, annealing at 55 °C for 30 s and extension at 72 °Cfor 45 s, and single extension at 72 °C for 10 min, and end at 4 °C. All samples were amplified in triplicate. The PCR product was extracted from 2% agarose gel and purified using the AxyPrepDNA Gel Extraction Kit (AXYGEN). Fluorescence quantification was performed with QuantiFluorTM-ST blue flourescence quantification system (Promege, USA).

Purified amplicons were pooled in equimolar amounts and paired-end sequenced on an Illumina NextSeq 2000 PE300 platform (Illumina, San Diego, USA) according to the standard protocols by Majorbio Bio-Pharm Technology Co. Ltd. (Shanghai, China).

The DNA library was constructed by TruSeqTM DNA Sample Prep Kit. After demultiplexing, the resulting sequences were quality filtered with fastp (v0.19.6) and subsequently merged using FLASH (v1.2.11). The high-quality sequences were denoised using DADA2 plugin in the Qiime2 (version 2020.2) pipeline with recommended parameters, which obtains single nucleotide resolution based on error profiles within samples. DADA2 denoised sequences are commonly referred to as amplicon sequence variants (ASVs). To mitigate the effects of sequencing depth on alpha and beta diversity measure, the number of sequence from each sample was rarefied to 20,000, resulting in an average Good's coverage of 97.90%. Taxonomic assignment of ASVs was performed using the Naive Bayes consensus taxonomy classifier implemented in Qiime2 and the SILVA 16S rRNA database (v138).

Bioinformatic analysis of the gut microbiota was carried out using the Majorbio Cloud platform (https://cloud.majorbio.com). Analysis of ASVs information included rarefaction analysis and estimation of alpha diversities, such as Ace, Chao, Sobs, and Coverage indices, which suggests the richness of observed species. These analyses were performed using Mothur (version v.1.30.2). Beta diversity, which estimates the similarity in community structure across samples, was evaluated through principal coordinate analysis (PCoA) based on Bray-Curtis distance using Vegan v2.4.3 package. Linear discriminant analysis (LDA) effect size (LEfSe) (http://huttenhower.sph.harvard.edu/LEfSe) was employed to identify significant differences in ASVs between two groups. The heatmap for key ASVs was visualized using MATLAB 2019b (The MathWorks, Inc., Natick, MA, USA). The network of correlation results was generated using Cytoscape v3.7.2.

### SCFAs analysis

Samples were transferred into 2 ml EP tubes and extracted with 1 ml H_2_O, followed by vortex mixing for 10 s. The samples were then homogenized in ball mill for 4 min at 40 Hz, then ultrasound treated for 5 min (incubated in ice water. This process was repeated 3 times. Tubes were centrifuged for 20 min at 5,000 rpm and 4 °C. The supernatant (0.8 ml) was transferred into a new 2 ml EP tubes; To this, 0.1 ml 50% H_2_SO_4_ and 0.8 ml of extracting solution (25 mg/L stock in methyl tert-butyl ether) were added as an internal standard, followed by vortex mixing for 10 s, oscillations in 10 min, and then ultrasonic treatment for additional 10 min (incubated in ice water). Tubes were then centrifuged for 15 min at 10,000 rpm and 4 °C. The samples were stored at −20 °C for 30 min before the supernatant was transferred into a fresh 2 ml glass vial for subsequent GC-MS analysis via SHIMADZU GC2030-QP2020 NX gas chromatography-mass spectrometer.

### Statistical analysis

All data are represented as mean ± standard deviation (SD). Statistical analyses were performed using SPSS 22.0 for Windows (SPSS Inc.). One-way Analysis of Variance (ANOVA) was used for comparisons between groups; The significance of differences in the measured α-diversities and β-diversity between two groups were assessed by Wilcoxon Rank-Sum Test with ggbox in MicrobiotaProcess. A threshold of LDA ≥ 2 was applied for both taxonomic and functional analyses to ensure a stringent detection of relevant group differences. Correlations and associations analyses between key ASVs and clinical parameters were calculated based on Spearman's rank correlation coefficients. The Benjamini and Hochberg method was adopted to calculate false discovery rate (FDR), thereby adjusting the significance of the correlations. A value of *P* < 0.05 was considered to indicate statistically significant difference.

## Results

### Characteristics of the participants involved in this study

In this study, clinical information and biological samples from a cohort of 31 individuals were gathered and subjected to comprehensive analysis. The participants were divided into two groups: MHT+ group (*n* = 17) and MHT– group (*n* = 14), as detailed in Table 1. Within the MHT+ cohort, all subjects were administered estrogen, either orally through Tibolone (*n* = 2), or transdermally via estradiol gel (*n* = 2), or orally through femostone (1/10 mg) (*n* = 13) in combination with progestogen, the duration of MHT was 0.5 to 7 years (2.23 ± 1.88 years). No significant discrepancies were observed between the two groups with respect to age, BMI, blood pressure, smoking and alcohol consumption, the presence of common chronic diseases and medication usage. Furthermore, no significant differences were detected in circulating levels of hemoglobin (Hb), white blood cells count (WBC), platelet count (PLT), fasting blood glucose (FBG), high-density lipoprotein cholesterol (HDL-c), low-density lipoprotein cholesterol (LDL-c), total cholesterol (TC), triglycerides (TG), and homocysteine (HCY) between the two groups.

**Table 1:** Demographic characteristics of the different groups.

**Demographic characteristics**	**MHT+ (*n* = 17)**	**MHT– (*n* = 14)**
Age (y)	51.53 ± 2.96	53.14 ± 1.66
Menopause age (y)	47.94 ± 2.70	49.29 ± 2.49
Menopause time (y)	3.59 ± 1.54	4.00 ± 2.57
Height (m)	1.58 ± 0.04	1.57 ± 0.05
Weight (m)	54.62 ± 5.49	52.93 ± 6.93
BMI (kg/m^2^)	21.93 ± 1.79	21.42 ± 1.99
Waistline (m)	0.77 ± 0.04	0.77 ± 0.07
Hipline (m)	0.92 ± 0.04	0.92 ± 0.06
Waistline/Hipline (m)	0.84 ± 0.03	0.85 ± 0.05
Systolic pressure (mmHg)	110.94 ± 10.02	117.57 ± 9.90
Diastolic pressure (mmHg)	69.65 ± 8.67	72.21 ± 5.56

Measurements are presented as mean ± SD.

Regarding sex steroid hormones, the serum levels of progestogen (Po) and testosterone (*T*) exhibited no significant difference between the two groups. However, the serum levels of estradiol (E2) were markedly higher in the MHT+ group relative to MHT– group, which was as expected. It is noteworthy that dydrogesterone, a progestogen widely used in MHT, does not show up in blood progestogen test. This explains why the serum progestogen levels in MHT+ were not found to be higher than those in the MHT- group despite their supplementation (see Table 2).

**Table 2:** Clinical indexes and hormone levels in the different groups.

**Clinical indexes**	**MHT+ (*n* = 17)**	**MHT– (*n* = 14)**
WBC ( × 10^9^)	5.34 ± 0.81	5.20 ± 1.38
Hb (g/L)	125 ± 7.37	125.57 ± 8.79
PLT ( × 10^9^)	224.53 ± 41.20	231.36 ± 41.69
FBG (mmol/L)	4.65 ± 0.37	4.81 ± 0.38
TC (mmol/L)	5.51 ± 0.89	5.30 ± 0.81
TG (mmol/L)	1.22 ± 0.46	1.61 ± 1.33
HDL-c (mmol/L)	1.73 ± 0.33	1.70 ± 0.40
LDL-c (mmol/L)	3.02 ± 0.88	2.76 ± 0.59
HCY (μmol/L)	11.85 ± 2.15	12.61 ± 2.44
E2 (pmol/L)^*^	206.29 ± 163.04	40.67 ± 19.83
T (nmol/L)	1.23 ± 0.51	1.06 ± 0.44
Po (nmol/L)	1.04 ± 0.95	0.58 ± 0.39

Measurements are presented as mean ± SD.

^*^Denotes a significant difference between two groups.

WBC, white blood cell count; Hb, hemoglobin; PLT, platelet count; FBG, fast blood sugar; TC, total cholesterol; TG, triacylglycerides; HDL-c, high density lipoprotein-cholesterol; LDL-c, low density lipoprotein-cholesterol; HCY, homocysteine; E2, estradiol; T, testosterone; Po, progestogen.

### Bone metabolic state

DXA scanner was employed to assess bone mineral content (BMC), bone mineral density (BMD). T-score, which serves as the primary diagnostic criterion for bone osteoporosis according to WHO criteria, is derived from BMD value. When comparing the two groups, no significant disparities were noted in the BMC of LS and FN; however, the BMD was significantly higher in MHT+ group compared to the MHT- group contributed to the protective effects of MHT.

Estrogen deficiency-induced osteoporosis is characterized by heightened bone turnover activity. In our study, markers such as CTX and PINP, which are indicative of osteogenic and osteoclastic/absorptive activity, were found to be significantly more elevated in the MHT- group compared to the MHT+ group. This suggests that individuals in MHT- group were in a state of high bone turnover. No significant differences were observed with respect to PTH, CT, Vitamin D levels between two groups (see Table 3).

**Table 3:** Results from DXA and BTMs.

**Parameters of bone condition**	**MHT+ (*n* = 17)**	**MHT– (*n* = 14)**
CTX (ng/mL)^*^	0.18 ± 0.09	0.46 ± 0.19
P1NP (ng/mL)^*^	32.28 ± 18.15	75.77 ± 19.01
PTH (ng/L)	44.36 ± 13.69	40.66 ± 12.48
CT (ng/L)	0.35 ± 0.85	0.36 ± 0.92
OC (ng/mL)^*^	24.66 ± 5.19	46.56 ± 15.89
VD2 (ng/mL)	1.31 ± 2.21	0.57 ± 0.61
VD3 (ng/mL)	22.56 ± 8.44	19.79 ± 5.81
VD (ng/mL)	23.87 ± 8.18	20.36 ± 5.50
LS-BMD (g/cm^2^)^*^	1146.97 ± 206.42	922.44 ± 177.56
LS-BMC (g)	51609.65 ± 9474.44	46443.07 ± 10436.54
FN-BMD (g/cm^2^)^*^	871.85 ± 113.52	767.19 ± 118.63
FN-BMC (g)	25669.29 ± 4143.50	23676.71 ± 4373.66

Measurements are presented as mean ± SD.

^*^Denotes a significant difference between two groups.

CTX, C-terminal telopeptide of type I collagen; P1NP, procollagen type I N propeptide; PTH, parathyroid hormone; CT, calcitonin; OC, osteocalcin; VD, vitamin D; BMD, bone mineral density; BMC, bone mineral content; LS, lumbar spine(L1-4); FN, femoral neck.

### Diversity comparison of gut microbiota from MHT+ and MHT– groups

**Figure 2:**
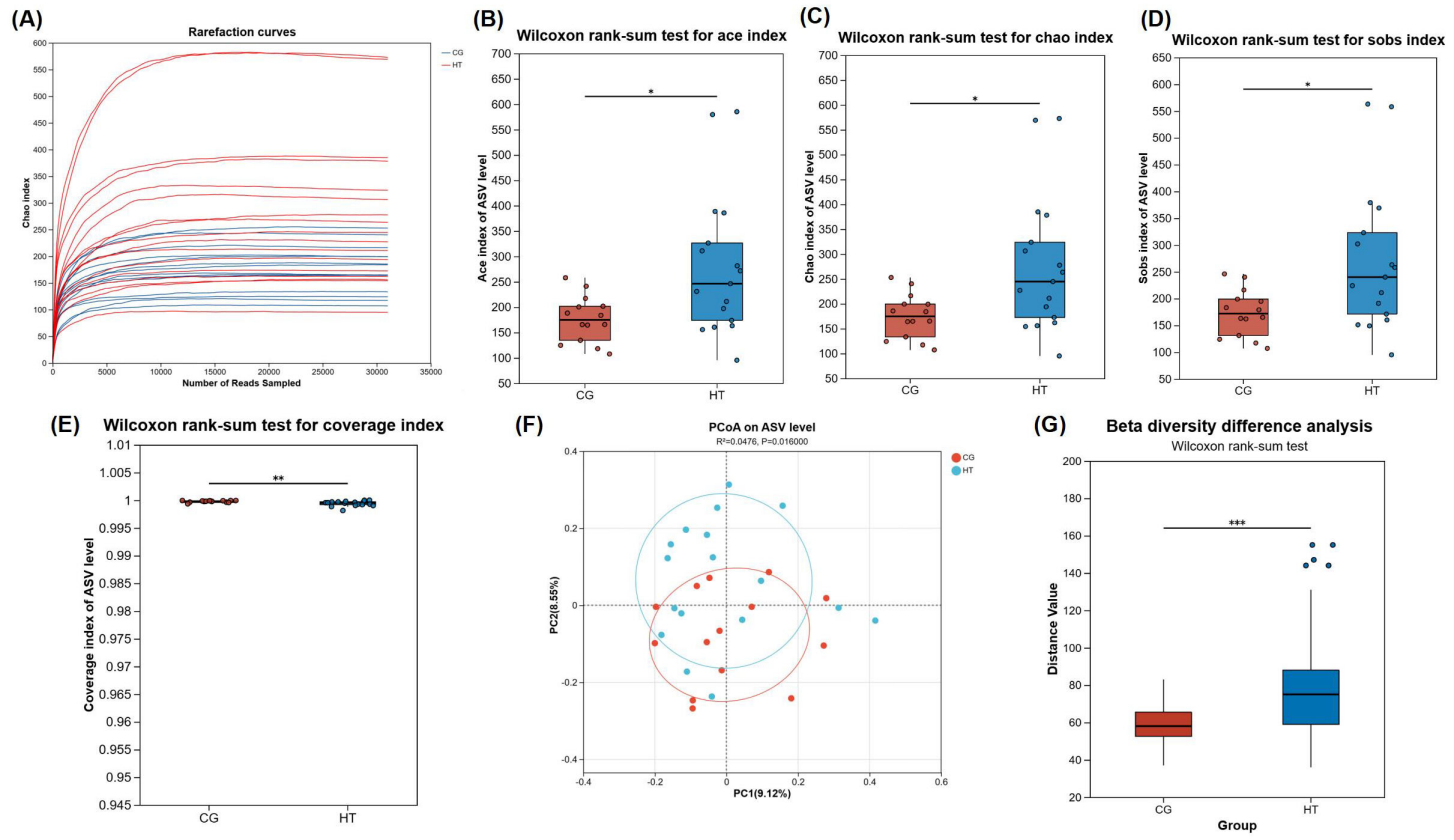
Diversity comparison of gut microbiota from MHT+ and MHT-groups. **(A)** Rarefaction curves. **(B)** Ace index. **(C)** Chao index. **(D)** coverage index. **(E)** Sobs index. **(F)** Bray-Curtis PCoA analysis. **(G)** Beta diversity difference analysis. CG, control group (MHT–); HT, hormone treatment (MHT+). *0.01 < *P* ≤ 0.05, **0.001 < *P* ≤ 0.01, ***P ≤ 0.001.

To ascertain the adequacy of the sequencing data and to characterize bacterial diversity, rarefaction analysis was performed, which revealed that each sample reached a plateau phase following a progressive increase (Figure 2A). The curve indicates that the sequencing data were robust sufficiently and could reflect the majority of the microbial composition in the samples reliably.

To determine if there were disparities between groups, microbial alpha diversity and beta diversity were conducted. Our results indicated a statistically significant enhancement in alpha diversity within the MHT+ group when compared to the MHT- group, as evidenced by their Ace, Chao, Sobs, and Coverage indexes (Figures 2B–E). Furthermore, a Bray-Curtis principal coordinate analysis (PCoA) was performed to examine beta diversity. The resultant PCoA plot showed an obvious separation between two groups, indicative of a pronounced disparity in the structure of the intestinal microbial communities (Figures 2F, G).

### Gut microbiota composition from MHT+ and MHT– groups

**Figure 3:**
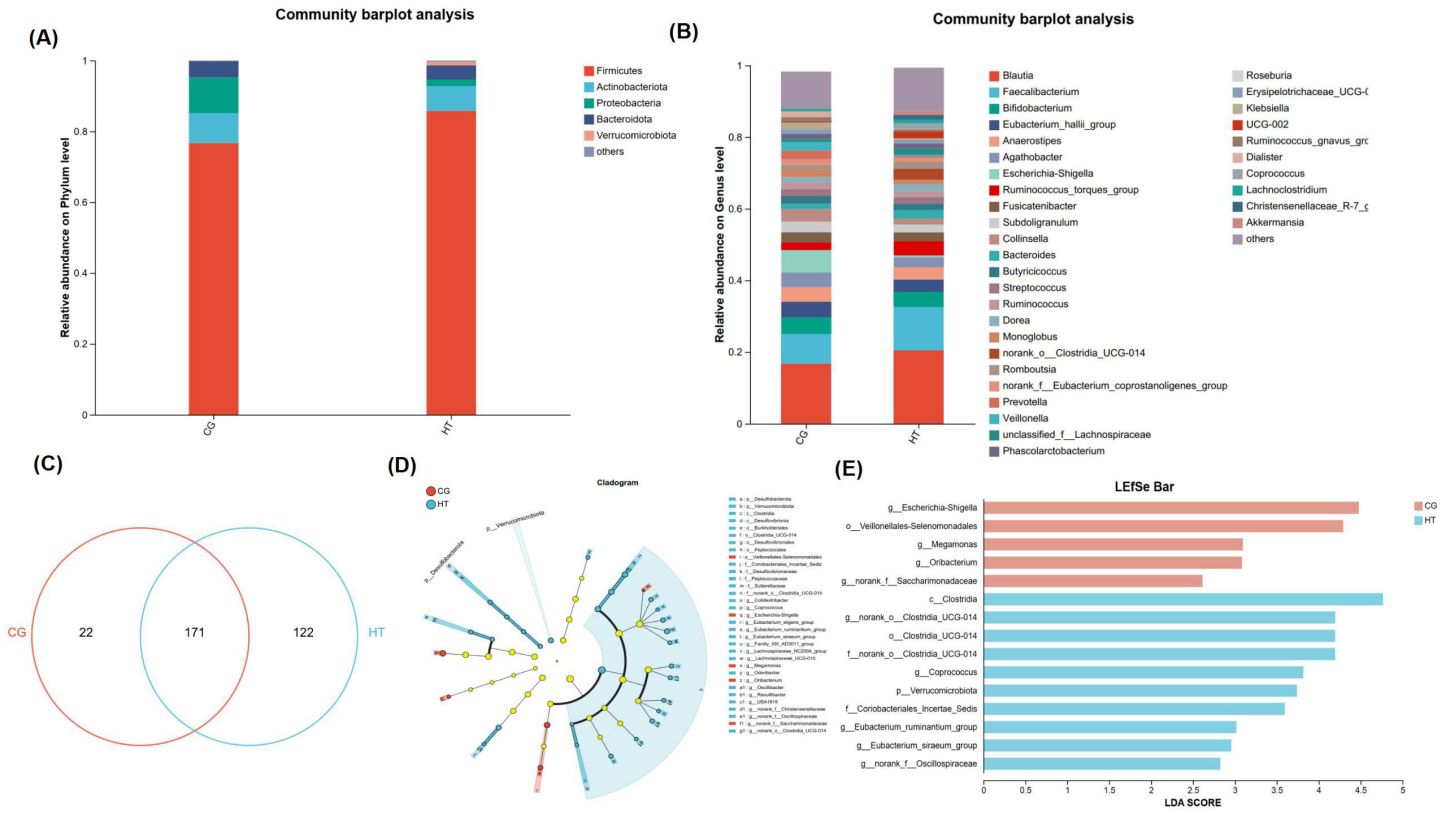
Gut microbiota composition from MHT+ and MHT- groups. **(A)** Gut microbiota at phylum level. **(B)** Gut microbiota at genus level. **(C)** Venn diagram for species composition analysis. **(D)** Bar chart of LDA value distribution in linear discriminant analysis effect size (LEfSe). **(E)** Evolutionary branch diagram of differential intestinal microbiota. CG, control group (MHT–); HT, hormone treatment (MHT+).

At the genus level, in addition to the 171 common species, the MHT+ group exhibited a significantly higher number of unique species (122) compared to the MHT– group (22). In order to explore potential discrepancies in gut microbiota composition based on women's hormone-replacement therapy status, we conducted a comparative analysis of the gut microbiota composition between the two groups at both the phylum, and genus level. Firmicutes was the most prevalent phylum in the microbiome of both groups. In MHT+ group, Actinobacteriota was the second most prevalent phylum, and followed by Bacteroidota and Proteobacteria. Conversely, in the MHT- group, Proteobacteria was the second most prevalent phylum, followed by Actinobacteriota and Bacteroidota. A notable increase in the abundance of Verrucomicrobiota and Desulfobacterota at the phylum level was observed in the MHT+ group relative to the MHT- group. The relative abundances of Proteobacteria was higher in the MHT– than MHT+, but not significant statistically. At the genus level, the relative abundances of Escherichia-Shigella was significantly higher in the MHT– group, whereas norank_o_Clostridia_UCG-014, Coprococcus, Eubacterium_ruminantium_group, norank_f_Oscillospiraceae, Eubacterium_eligens_group, Family_XIII_AD3011_group, Eubacterium_siraeum_group, UBA 1819, Lachnospiraceae_UCG-010 were significantly more abundant in the MHT+ group (Figures 3A–C). Further, linear discriminant analysis effect size (LEfSe) demonstrated an obvious difference existing between the two groups. An increased abundance of Escherichia-Shigella, Veillonelales-Selenomonadales, Megamonas, Oribacterium, norank_f_Saccharimonadaceae was observed in the MHT– group, while Clostridia, norank_o_Clostridia, Coprococcus, Verrucomicrobiota, Coriobacteriales_Incertae_Sedis, Eubacterium_ruminantium_group, Eubacterium_siraeum_group, norank_f_Oscillospiraceae were more abundant in the MHT+ group (Figures 3D, E).

### Correlations between the bacteria and clinical indexes

The relationships among various bacterial species, BTMs, and other clinical parameters were assessed by Spearman correlation analysis. The findings revealed that Clostridia_UCG-014 and Coprococcus exhibited a significant negative correlation with CTX and P1NP, suggesting a potential protective role in bone health. The correlation strength was moderate (−0.06 < *R* < −0.04). Additionally, these genera showed a positive correlation with serum E2 levels and BMC as well as BMD at the LS and FN, although not statistically significant. Other genera including Lachnospiraceae_NC2004_group and Eubacterium eligens group demonstrated similar trends in their correlations with these indexes, albeit with weaker correlation strength.

**Figure 4:**
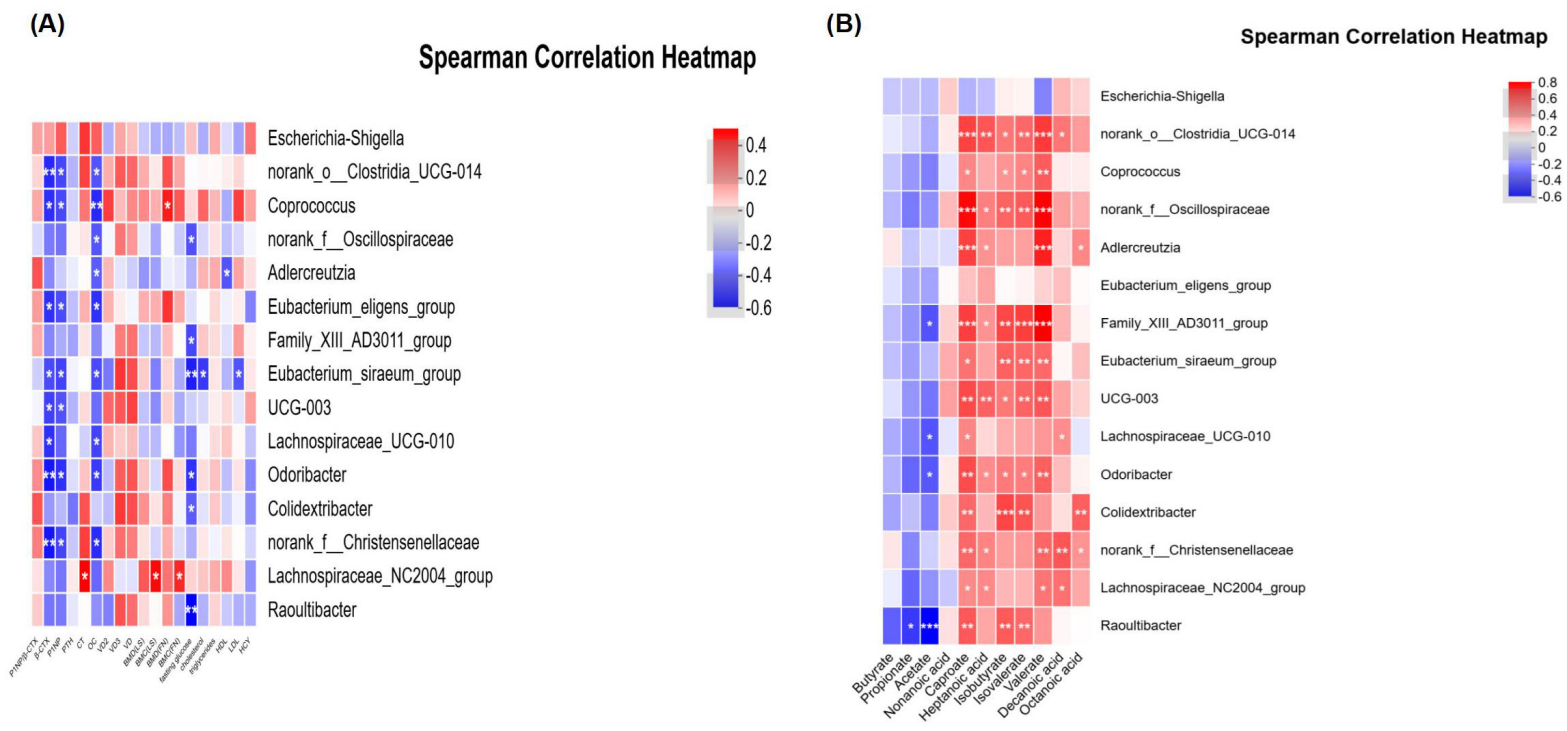
Correlations between the bacteria and other indexes. **(A)** Relationship between intestinal flora and clinical indexes. **(B)** Relationship between intestinal flora and SCFAs. CG, control group (MHT-); HT, hormone treatment (MHT+). *0.01 < *P* ≤ 0.05, **0.001 < *P* ≤ 0.01, ***P ≤ 0.001.

In contrast, Escherichia-Shigella, which was significantly associated with infectious diseases, displayed a negative correlation with serum E2 and BMC, BMD at LS and FN, while positively correlating with CTX and P1NP (although not significantly). Another genera, Odoribacter, was strongly and negatively correlated with CTX and P1NP (*P* < 0.05), and correlated with LS BMD and FN BMD positively. A similar trend was observed for Colidextribacter. With the exception of Escherichia-Shigella, most of these genera also exhibited a strong association with serum E2 levels, regardless of statistical significance (Figure 4A) (Supplementary materials 1, 2).

### SCFAs profiles and correlations with bacteria

No significant differences in SCFAs were observed between the two groups, suggesting a limited role in bone metabolism (see Table 4). Several genera, including Clostridia_UCG-014, Coprococcus, Oscillospiraceae, Family_XIII_AD3011_group, Eubacterium_siraeum_group, UCG-003, Lachnospiraceae_UCG-010, Colidextribacter, Christensenellaceae, and Lachnospiraceae_NC2004_group (all belonging to the phylum Firmicutes), as well as Adlercreutzia and Raoultibacter (phylum Actinobacteriota), and Odoribacter (phylum Bacteroidota), were found to be positively and moderately associated with various SCFAs (*P* < 0.05). In contrast, Escherichia-Shigella demonstrated a negative correlation with the majority of SCFAs, although this association did not reach statistical significance (see Figure 4B) (Supplementary materials 3, 4).

**Table 4:** Concentration of SCFAs in two groups (mg/L).

**SCFAs**	**MHT–**	**MHT+**
Butyrate	52.48 ± 40.04	38.77 ± 20.08
Propionate	42.82 ± 20.88	28.9 ± 10.91
Acetate	55.32 ± 19.43	50.04 ± 12.92
Nonanoic acid	0.14 ± 0.03	0.13 ± 0.04
Caproate	0.21 ± 0.51	0.31 ± 0.62
Heptanoic acid	0.02 ± 0.03	0.06 ± 0.15
Isobutyrate	3.45 ± 3.66	5.18 ± 4.44
Isovalerate	3.44 ± 4.53	5.24 ± 5.48
Valerate	3.56 ± 5.59	5.59 ± 4.64
Decanoic acid	0.04 ± 0.06	0.02 ± 0.02
Octanoic acid	0.05 ± 0.09	0.04 ± 0.02

Measurements are presented as mean ± SD.

## Discussion

The gut microbiota, by modulating the host's immune, metabolic, and endocrine systems, participates in various host physiological activities, exerting a broad spectrum of effects within the body and is considered a key factor influencing human health. As we know, intestinal epithelial cells are sealed by tight junction proteins (TJs) including ZO-1 and occludin, dysbiosis of gut microbiota can alter the expression and distribution of TJs, coupled with the disruption of the structural and functional integrity of the intestinal barrier. The permeability altering of the intestinal barrier facilitates the translocation of gut microbes and their metabolites, thereby triggering a local inflammatory state within the intestines. This inflammatory state, once entering the systemic circulation, can further amplify its impact, inducing systemic immune system imbalance and causing inflammatory responses in other tissues or organs, thereby leading to damage to distant target organs. This is one of the main mechanisms of the “gut-bone axis”, which is involved in aging (10, 11). Oxidative stress contributes to the development of osteoporosis by enhancing the breakdown of bone by osteoclasts and reducing the formation of new bone by osteoblasts. This process involves complex signaling pathways, including NF-κB, PI3K/Akt, and Wnt/beta-catenin (12, 13). Additionally, T cells, a type of immune cell, play a role in this process by influencing the balance between different T cell subsets (Th17 and Treg) and the production of various signaling molecules, or cytokines. Interestingly, consuming certain beneficial bacteria, known as probiotics, can help reduce inflammation throughout the body and in the bones, which is often linked to changes in how permeable the intestines are. This, in turn, helps to preserve bone density (14).

The gut microbiota can promote the absorption of calcium and vitamin D and produce serotonin and SCFAs, all of which can affect bone formation and resorption (15). In addition, extracellular vesicles (EVs) produced by the gut microbiota can cross the intestinal epithelial barrier to reach distant target tissues and regulate the function of distant target organs (16). This new mechanism has been found to be involved in the well-known protective effect of the beneficial bacterium including Akkermansia on osteoporosis (17, 18). Observations and comparisons of the gut microbiota in people with different bone mass and hormone levels have been reported (19), and the activation of estrogen and the gut microbiota interact with each other, suggesting a close connection among bone metabolism, hormones and the gut microbiota (9, 20, 21). The roles of sex steroids, gut fora and gut dynamics are also interrelated. Estrogens and androgens play important regulatory roles in gut motility and psychological conditions, possibly through modulating the gut-brain axis (22), while the acceleration or slowdown of intestinal motility will affect the reproduction of suitable bacterial species and change the composition and function of the microbiota. Meanwhile, dysbiosis of the gut microbiota and their metabolites can Induce Intestinal motility disorder, affect the contraction of intestinal smooth muscle and the transmission (23).

Hormone replacement therapy aims to protect bone mass and cardiovascular health by supplementing estrogen in postmenopausal women for a long term. Its protective effect on the cardiovascular system has been confirmed to be related to the gut microbiota (24), and changes in the gut microbiota also occur during the treatment of osteoporosis with various traditional Chinese and western medicines (25).

This study enrolled a homogeneous cohort of participants, exhibiting no statistically significant disparities in geographical demographics, dietary patterns, menopausal duration, chronological age, body mass index, fasting blood glucose, lipid profiles, blood pressure, and inflammatory status indicated by leukocyte cell count. This stringent participant selection criterion effectively minimized the influence of other factors on the gut microbiota. DXA assessments revealed a lack of difference in BMC and a small difference in BMD, which may attribute to the relatively short time since menopaus among the enrolled females (average menopausal duration was < 4 years). Nonetheless, the observed discrepancies in skeletal metabolism biomarkers, including P1NP, CTX, and OC, underscored that the bone metabolism status of women under different treatment conditions varies. MHT exerts a beneficial effect on bone density preservation through long-term administration. This further emphasized the focus of this study on the role of gut microbiota in the process of bone metabolism by MHT, while previous studies mostly reported comparisons of the microbiota after bone mass differences had occurred. This study demonstrated a significant impact of hormone replacement therapy on the intestinal microbiota of women, significantly increasing the diversity of the intestinal microbiota. The ratio of Bacteroidetes to Firmicutes is an important indicator of the balance of the human microbiota. From the composition structure of the microbiota, it can be seen that the ratio of the two groups, MHT+ and MHT–, is different, with a higher proportion of Firmicutes in the MHT+ group. LEfSe analysis indicated that the relative abundance of Verrucomicrobiota was higher in the MHT+ group. Akkermansia, which belongs to this phylum and has significant effects in immunity, anti-aging, and anti-cancer, indeed had a higher abundance in the MHT+ group, but the difference was not statistically significant.

When analyzing the dominant genera with statistical differences between the two groups, we found that both Escherichia-Shigella and Megamonas were present in the MHT- group simultaneously. Coincidentally, both are closely related to inflammatory responses. Escherichia-Shigella is the most common pathogen causing bacillary dysentery in humans (26); Megamonas is also closely associated with local intestinal inflammatory diseases, colorectal cancer, systemic obesity, ankylosing spondylitis, and autism spectrum disorders (27). In polycystic ovary syndrome, a female endocrine and metabolic disorder, the abundance of Escherichia-Shigella also increased, suggesting a possible link with hormonal imbalance (28). Correlation studies have confirmed that this bacterium is negatively correlated with estrogen levels and bone density, while positively correlated with increased bone metabolism indicators (*P* < 0.05).

The abundance of Clostridia also varies among different bone mass groups, being higher in the normal bone density group. This finding is consistent with previous reports (29), indicating significant differences in component abundance. LDA analysis suggests that Clostridia abundance is higher in the MHT+ group and positively correlated with bone mass, serum estradiol, and vitamin D levels, while negatively correlated with bone metabolism indicators such as P1NP and CTX moderately (*P* < 0.05). Other genera with similar correlations include Coprococcus, Eubacterium, Colidextribacter, and Lachnospiraceae_NC2004_group. The protective effect of MHT on bone mass is likely the result of the combined action of multiple microbial groups. These genera may play important bone-protective roles under the regulation of estrogen in MHT and all belong to the Clostridia class of the Firmicutes phylum. Coprococcus is an important genus in the human gut, capable of actively fermenting carbohydrates and is a significant producer of butyric acid among intestinal microorganisms (30). Bacteria of the Coprococcus genus help suppress immune responses in the body and have been found to be associated with constipation, language development in children, depression, and chronic fatigue (31, 32). In terms of bone metabolic disease, Coprococcus and Blautia potentially perturb glucose homeostasis via the generation of SCFAs, thereby influencing osteoclastic metabolism and contributing to the pathogenesis of tibial dyschondroplasia (33).

We also conducted SCFAs analysis and correlation analysis with the intestinal microbiota. The results indicated that many genera positively correlated with bone protection are also associated with the production of certain SCFAs. Adlercreutzia belongs to the Firmicutes phylum and Bifidobacteriaceae family. They can metabolize and ferment in the human gut, producing xylose, arabinose, and SCFAs such as acetic acid, propionic acid, and butyric acid (34). It plays an important role in regulating the host's intestinal environment, immune status, promoting nutrient absorption, and maintaining intestinal health. In this study, its abundance in the MHT+ group was nearly five times that in the MHT– group, suggesting it may also be a factor in the protective effect of MHT on menopausal women. Although the abundance of many beneficial bacteria related to butyric acid production significantly increased in the MHT+ group, the butyric acid production in the MHT+ group was not higher than that in the MHT- group. This may be related to the relatively low proportion of other major butyric acid-producing bacteria such as Butyricicoccus in the MHT+ group.

This study explored the impact of MHT on the gut microbiota in postmenopausal women and its relationship with bone metabolism, laying a foundation for exploring the role of the gut microbiota in bone mass protection. However, it still has certain limitations. Firstly, the number of subjects enrolled in the two groups was relatively small, which might compromised the precision and statistical power of the results. Secondly, although this study included different MHT options, the effects of different MHT options on the gut microbiota may also vary, which requires further investigation in subsequent studies. Thirdly, this study only conducted a correlation study between bacterial genera and bone metabolism indicators, without further verification and exploration of the underlying specific mechanisms, which awaits validation through animal experiments.

This study contributes to a better understanding and promotion of MHT and is of great significance in exploring new approaches to prevention and treatment of osteoporosis.

## Data Availability

Due to the sensitive nature of the data collected for this study, which includes potentially identifying patient information, the data are not publicly available. However, the data are available from the corresponding author upon reasonable request, subject to the approval of an institutional ethics committee and a data use agreement is acceptable.
